# Enriching captivity conditions with natural elements does not prevent the loss of wild‐like gut microbiota but shapes its compositional variation in two small mammals

**DOI:** 10.1002/mbo3.1318

**Published:** 2022-09-28

**Authors:** Adam Koziol, Iñaki Odriozola, Lasse Nyholm, Aoife Leonard, Carlos San José, Joana Pauperio, Clara Ferreira, Anders J. Hansen, Ostaizka Aizpurua, M. Thomas P. Gilbert, Antton Alberdi

**Affiliations:** ^1^ Center for Evolutionary Hologenomics, Globe Institute University of Copenhagen Copenhagen Denmark; ^2^ Biodonostia Health Research Institute Donostia‐San Sebastian Spain; ^3^ CIBIO—Centro de Investigação em Biodiversidade e Recursos Genéticos, InBIO Laboratório Associado Universidade do Porto Vila do Conde Campus de Vairão Portugal; ^4^ Animal Ecology, Institute for Biochemistry and Biology University of Potsdam Potsdam Germany; ^5^ University Museum, Norwegian University of Science and Technology Trondheim Norway

**Keywords:** 16S, captivity, diversity loss, gut‐microbiome, host‐associated microbiota, non‐model organism

## Abstract

As continued growth in gut microbiota studies in captive and model animals elucidates the importance of their role in host biology, further pursuit of how to retain a wild‐like microbial community is becoming increasingly important to obtain representative results from captive animals. In this study, we assessed how the gut microbiota of two wild‐caught small mammals, namely *Crocidura russula* (Eulipotyphla, insectivore) and *Apodemus sylvaticus* (Rodentia, omnivore), changed when bringing them into captivity. We analyzed fecal samples of 15 *A. sylvaticus* and 21 *C. russula*, immediately after bringing them into captivity and 5 weeks later, spread over two housing treatments: a “natural” setup enriched with elements freshly collected from nature and a “laboratory” setup with sterile artificial elements. Through sequencing of the V3–V4 region of the 16S recombinant RNA gene, we found that the initial microbial diversity dropped during captivity in both species, regardless of treatment. Community composition underwent a change of similar magnitude in both species and under both treatments. However, we did observe that the temporal development of the gut microbiome took different trajectories (i.e., changed in different directions) under different treatments, particularly in *C. russula*, suggesting that *C. russula* may be more susceptible to environmental change. The results of this experiment do not support the use of microbially enriched environments to retain wild‐like microbial diversities and compositions, yet show that specific housing conditions can significantly affect the drift of microbial communities under captivity.

## Graphical abstract

We assessed how the gut microbiota of two wild‐caught small mammals, *Crocidura russula* (greater white‐toothed shrew, Eulipotyphla, insectivore) and *Apodemus sylvaticus* (wood mouse, Rodentia, omnivore), changed when bringing them into captivity and housing them under different conditions. Our results showed that gut microbial diversity was significantly reduced when bringing wild animals into captivity regardless of housing conditions. However, while diversity was lost, the temporal development of the gut microbiome was very different between treatments, particularly in *C. russula*.

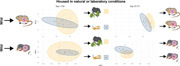

## 1 INTRODUCTION

The study of host‐microbiota interactions has become integral to our understanding of animal health, ecology, and evolution (Nyholm et al., 2020). Due to the complexity of gut microbial communities, and their sensitivity to environmental factors, captivity experiments (both using laboratory and wild animals) have proven essential for detecting and measuring the detailed interactions between animals and microorganisms (Hird, 2017; Rosshart et al., 2019; Shinohara et al., 2019). Such set‐ups enable the controlling and limiting of experimental factors that may influence the measured outcome, including host genetic variation (Bonder et al., 2016), developmental stage (Arrieta et al., 2014), and social interactions (Raulo et al., 2021), as well as environmental factors such as temperature (Sepulveda & Moeller, 2020), humidity (Rosenbaum et al., 2009) and diet (Bibbò et al., 2016; Martínez‐Mota et al., 2020; Maurice et al. 2015; Morrison et al., 2020). However, simplified captive environments can also modify the microbiota in a variety of ways, by reducing its diversity in comparison to that of wild communities, or recruiting new bacteria that are not found in wild populations (Alberdi et al., 2021). Such changes can differ significantly across species (Kohl et al., 2014), and might also decouple the optimal animal‐microbiota balance dropping host fitness (Rosshart et al., 2017). Hence, adequate assessment of all experimental variables relating to gut microbiota dynamics is important, as deviations from healthy biotic states can lead to erroneous experimental outcomes (Beura et al., 2016; Kinross et al., 2011).

In light of such limitations and biases, researchers are actively seeking strategies to firstly maintain the original gut microbial communities of wild animals once they have been moved to captivity, and secondly, modify the gut microbiota of laboratory animals to resemble that of their wild counterparts (Rosshart et al., 2019). Since the diet is one of the factors that conditions gut microbial communities, attempts have been made to employ dietary interventions to achieve these goals (Martínez‐Mota et al., 2020). An alternative strategy that has been explored is the introduction of microbes through non‐dietary related environmental sources, with some studies demonstrating there can be a significant positive effect on the gut microbiome (Liu et al., 2021; Weinstein et al., 2021; Zhou et al., 2016). While the inclusion of environmental microbes in captivity experiments has been assessed to have positive outcomes, no studies have addressed this in the context of a management tool, thus making it important to assess the value of microbially enriching the environment used in captivity experiments.

To explore a new potential way to help captive animals retain wild‐like gut microbiotas, we studied whether enriching captivity housing conditions with natural elements (while maintaining diet as a constant) contributes to the retention of the original (precaptivity) gut microbial community, as proxied by fecal samples, of animals captured in the wild. We carried out our experiment on two widespread non‐model small mammals with differing evolutionary history and ecology: the European wood mouse *(Apodemus sylvaticus*—AS, order Rodentia, omnivorous diet) and the greater white‐toothed shrew (*Crocidura russula*—CR, order Eulipotyphla, insectivorous diet). The animals were kept in captivity for 5 weeks under two different treatments: a “Natural” setup containing enrichment elements freshly collected from nature, and a “Laboratory” setup containing artificial enrichment elements. We analyzed variations in the gut microbiota from various perspectives: (i) the change in alpha diversity to assess if nature‐enriched conditions contributed to maintaining wild‐like gut microbial diversity; (ii) the change in beta diversity between time points and within individuals to explore whether nature‐like conditions maintained a composition more similar to the original wild‐like community, and (iii) the interacting effects of treatment and time on bacterial community composition to explore if the community changed in different directions over time (i.e., if the microbial community took different temporal trajectories) under the contrasting captivity conditions. Using the Hill numbers framework, we calculated neutral and phylogenetic diversity and dissimilarity indices at multiple orders of diversity (Chao et al., 2014). This allowed us to disentangle the contribution of closely versus distantly related bacteria and rare versus common bacteria to the variations between time points and treatments for each host species.

## 2 METHODS

### 2.1 Animal trapping and collection

Adult AS and CR were collected across the Northern Iberian Peninsula, Europe (43.2 N, 2.2 W), from June to August 2019 (due to trapping success) over 11 field sites. Animals were trapped using Sherman traps over 3 days at each site and checked every 12 h. Traps were cleaned between locations and the baits used were a mixture of oats and tuna and a small wedge of apple. Upon successful detection, each animal was transferred into a plastic bag for species and sex identification. Maturity of the animal was confirmed with morphometrics (e.g., body weight and length) and pelage for both species, any individuals which did not meet adult criteria or which were pregnant/lactating were excluded and released. Individuals were then individually placed into a small, microisolator cage for transfer to the ZIBA animal experimentation facilities in Zarautz, Basque Country, Spain.

### 2.2 Processing and identification of shrews and mice

Before experimental inclusion, animals were checked for any signs of serious distress or ailment, and if deemed healthy to continue, an initial fecal sample (~50 mg) was collected upon arrival at the experimentation facility. Each animal was then anesthetized over a heated mat using 2% isoflurane, to allow the subcutaneous injection of a Mini HPT10 radio frequency identification chip (Biomark) into the nape of the neck for subsequent individual identification. Each individual was monitored for 5 minutes for any adverse effects before being transported into the corresponding housing enclosure.

### 2.3 Housing conditions and experimental design

**Figure A1:**
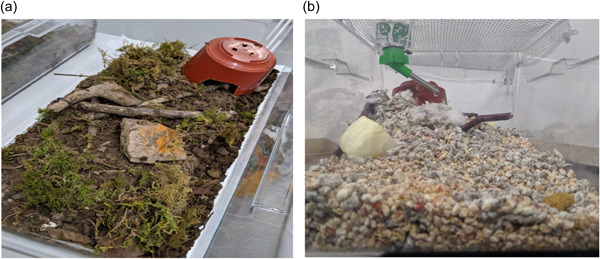
Examples of housing conditions (a) natural conditions and (b) laboratory conditions

Animals were cohoused with conspecifics of the same sex in groups of 4–5 individuals in 840 cm^2^ polycarbonate cages (Unno Type III, 38.2 × 22.0 cm). Cages were randomly assigned to two different environmental enrichment conditions, either Natural conditions (herein NC) or Laboratory conditions (herein LC). Although the cages used in both conditions contained similar three‐dimensional enrichment structures, the structures themselves were created using either natural or artificial elements, respectively (Appendix A: Figure A1). NC involved the use of natural elements freshly collected from the habitats in which the animals were trapped; specifically, the soil was used as bedding, moss was provided as nesting material, and sticks and stones were used as enrichment elements. All enrichment materials for NC were collected from natural areas of low human encroachment and hence are unlikely to consist of any human features. LC included paper and wood bedding, cotton as nesting material, and 3D‐printed plastic sticks and stones as enrichment elements. For both treatments, cages were cleaned and materials replaced or sterilized every week. In NC soil, nesting and enrichment items were replaced with fresh materials, while in LC fresh bedding and nesting materials were added and the enrichment materials cleaned and sterilized. With regard to diet, AS were fed a standard chow diet, while CR were fed a feed containing rice and chicken. Both diets were maintained unchanged across the whole experimental period. Animals were kept under a strict 12 h night and day cycle, and routine cage cleaning was performed each week (replacement of bedding and nesting material), access to food was *ad libitum*, and food was changed daily. Environmental conditions were kept constant with an average humidity of 70%, temperature of 22°C, and 60 revolutions of air per minute by keeping the animals in an HPP 750 LIFE climate controller chamber (Memmert).

### 2.4 Fecal collection

We sampled from AS (*n* = 15) and CR (*n* = 21) housed in four and five cages, respectively. The experiment was sex‐biased towards male individuals (*n*_AS_ = 13, *n*_CR_ = 13), due to uneven capture success. Animals were split into NC (*n*_AS_ = 9, *n*_CR_ = 12, cages_AS_ = 2, cages_CR_ = 3) and LC (*n*_AS_ =  6, *n*_CR_ = 9, cages_AS_ = 2, cages_CR_ = 2) housing treatments. Fresh feces were collected from each individual immediately upon arrival at Time point 0 (herein T0; approximately 30 min –1 h after arrival to the laboratory) and day 35 (herein T1). To do so, animals were isolated into a separate sterile housing container and upon defecation, the fecal pellets (~50 mg) were collected and stored in 500 μl of DNA/RNA shield (Zymo), left at room temperature for 1 hour, and then transferred to −20°C for long‐term storage until DNA extraction.

### 2.5 DNA extraction and metabarcoding

DNA was extracted using a Zymo QuickDNA Fecal/Soil Microbe 96 kit (Zymo) according to manufacturer's guidelines, eluted in 50 μl of elution buffer, and immediately stored at −20°C. This involved an initial quality check for DNA concentration using a Tapestation high sensitivity kit (Agilent). Immediately after, amplification of the V3–V4 region of the 16S recombinant RNA (rRNA) gene was performed using the primers 341F:ACTCCTACGGGAGGCAGCAG (Herlemann et al., 2011) and 806R:GGACTACHVGGGTWTCTAAT (Takai & Horikoshi, 2000) using fusion tags with unique indices for downstream identification. PCR was performed in a total volume of 50 μl consisting of 25 μl of NEB Phusion® high‐fidelity PCR master mix, 4 μl of reverse and forward fusion tag primers, 30 ng of DNA extract, and ddH_2_0 up to 50 μl. PCR conditions consisted of an initial denaturation step of 98°C for 3 min, 30 cycles of denaturation at 98°C for 45 s, annealing at 55°C for 45 s, elongation at 72°C for 45 s, and lastly a final hold at 72°C for 7 min. After amplification, the PCR products were purified using Ampure beads (Agencourt) to remove small fragments and impurities. Samples were then quality and concentration checked by a Tapestation on a high sensitivity chip (Agilent) and pooled equimolar before sequencing 300 PE on a HiSeq. 2500 (Illumina) using services from BGI. Negative extraction controls were included throughout all stages of the process to control for cross‐contamination.

### 2.6 Bioinformatics and data analysis

Paired‐end reads were first demultiplexed on unique fusion tag combinations. Immediately following this we quality‐filtered the demultiplexed reads (Q > 20) using AdapterRemoval 2.3.1 (Schubert et al., 2016), and primers were removed using Cutadapt 2.10 (Martin, 2011). Low‐quality reads were removed or trimmed using the filterAndTrim function implemented in DADA2 (Callahan et al., 2016). Error pattern learning and denoising of the data set were also performed using the DADA2 algorithm using default parameters (Callahan et al., 2016). Chimera removal was then performed before the generation of an ASV table consisting of ASV read counts for each sample. Reads were abundance‐filtered across samples by a relative abundance of 0.01% to remove singletons and other reads that may exist due to sequencing or PCR artifacts. Taxonomy assignment was then performed by the naïve Bayesian classifier implemented in DADA2 against the SILVA 16S taxonomy database (v138). Alignment of ASV sequences was performed using Clustal Omega (Madeira et al., 2019) and subsequently, a phylogenetic tree was built in Iqtree (Minh et al., 2020). ASVs were filtered using the R package decontam (Davis et al., 2018) to detect relevant contaminants based on the prevalence algorithm.

### 2.7 Diversity and compositional modeling

Gut microbiota diversity and compositional analyses were based on the Hill numbers framework. Specifically, we computed both neutral and phylogenetic diversities of orders of diversity (*q* value) 0, 1, and 2 using the R package Hilldiv (Alberdi, 2019). Neutral metrics do not account for the degree of relatedness among ASVs, while phylogenetic metrics consider the phylogenetic correlations among ASVs when computing diversity. Differences between both dimensions of diversity metrics (neutral and phylogenetic) therefore provide insights into whether diversity variation is driven by phylogenetically close or distantly related taxa. The different orders of diversity assign different weights to the ASVs when computing diversity. A *q* value of 0 does not consider relative abundances but only the presence or absence of ASVs. At a *q* value of 1, ASVs are weighted according to their relative abundances. A *q* value of two overweighs abundant ASVs with respect to nonabundant ones. Comparisons between orders of diversity therefore yield information on how the evenness of ASV distribution within samples affects diversity estimation. Beta diversity between the two sampling time points (i.e., before and after the captivity period) was also measured in terms of Hill numbers by computing the Sørensen‐type turnover. Similarly, Sørensen‐type turnover derived from all sample pairs in the data set was used to assess the directional effect of treatment and time points in gut microbial composition (Alberdi & Gilbert, 2019; Chao et al., 2014).

Linear mixed‐effect models, as implemented in the R package nlme (Pinheiro et al., 2017), were employed to assess the change in alpha diversity and beta diversity in response to experimental treatments on the gut microbiota across all individuals. In total, eight linear mixed models (Table 1) were fitted for each combination of species (i.e., AS and CR), diversity metric (i.e., neutral and phylogenetic), and also by diversity scale (i.e., alpha and beta diversity metrics). For alpha diversity models we included as fixed explanatory variables the *q* value (categorical factor with three levels: “0,” “1,” and “2”), treatment (categorical factor with two levels: “natural” and “laboratory”), time (categorical factor with two levels: “T0” and “T1”) and their interactions. As several individuals were kept in each cage, and several diversity metrics were calculated from each sample, a random effect of the form “~1|Cage/Individual_ID/Sample_ID” was included in the models. Beta diversity was measured as the compositional change from T0 to T1 within each individual, hence, only treatment, *q* value, and their interaction were used as fixed factors and, a random effect of the form “~1|Cage/Individual_ID” was included. Linear mixed models were checked for assumptions of homoscedasticity and normality of residuals and, where assumptions were violated (e.g., alpha diversity metrics), the response variables were log‐transformed. Model complexity was reduced by dropping the nonsignificant interactions between the fixed effects using likelihood ratio tests between nested models. Regardless of their significance, all main effects as well as the random effects were retained in the models as structural parts of the experimental design.

**Table 1:** Final linear mixed models

Model parameters			
Model name	Neutral/phylogenetic	Alpha/beta	Model equation
Crocidura1	Neutral	Alpha	diversity~time + treatment + *q* value, random = ~1|Cage/Mouse_ID/Sample_ID
Crocidura2	Phylogenetic	Alpha	diversity~time + treatment + *q* value, random = ~1|Cage/Mouse_ID/Sample_ID
Crocidura3	Neutral	Beta	dissimilarity~treatment:*q* value, random = ~1|Cage/Mouse_ID
Crocidura4	Phylogenetic	Beta	dissimilarity~treatment:*q* value, random = ~1|Cage/Mouse_ID
Apodemus1	Neutral	Alpha	diversity~time + treatment + *q* value, random = ~1|Cage/Mouse_ID/Sample_ID
Apodemus2	Phylogenetic	Alpha	diversity~time + treatment × *q* value, random = ~1|Cage/Mouse_ID/Sample_ID
Apodemus3	Neutral	Beta	dissimilarity~treatment:*q* value, random = ~1|Cage/Mouse_ID
Apodemus4	Phylogenetic	Beta	dissimilarity~treatment:*q* value, random = ~1|Cage/Mouse_ID

The temporal change in gut microbial composition (i.e., beta diversity between time points) may happen following independent trajectories in each individual, or directionally, following a specific trajectory across all individuals. To test the null hypothesis of no directional changes in microbiome composition from the transition of wild (T0) to day 35 (T1) we used PERMANOVA (Anderson, 2017) on pairwise dissimilarity matrices based on Sørensen‐type turnover (neutral and phylogenetic, and combining different *q* values) using the function adonis2 in the R package “vegan” (Oksanen et al., 2020). We fitted two PERMANOVA models per type of dissimilarity matrix, one for each host species with the form adonis2(microbiome ~  treatment × time, strata = Individual_ID). A significant treatment × time interaction would indicate that the enrichment with natural elements led to different temporal trajectories in community composition. The magnitude of effects was quantified using the adjusted *R* ^*2*^ and the microbiome composition visualized using NMDS (Kruskal, 1964).

To identify the bacterial genera most severely affected by captivity conditions in each housing condition, we analyzed the data through hierarchical modeling of species communities (HMSC) at the genus level (Warton et al., 2015), as implemented in the R package HMSC (Tikhonov et al., 2020). HMSC is a hierarchical model constructed in the generalized linear model framework using Bayesian inference. Four models were fitted, separately for each of the two species and the NC and LC. As the data were zero‐inflated, we applied a hurdle model (zero‐altered model) (Rose et al., 2006). This type of model consists of two parts, one modeling the presence‐absence of species and the other modeling abundance conditional on presence. To fit the first model, we transformed all nonzero values in the data set into one, to create a presence‐absence matrix. We applied a binomial model with a probit link function to each genus. The second model looks at abundances conditional on presences (scaled to mean zero and unit variance). We transformed zeros to missing values, and kept all nonzeros in their values, we then fitted the log‐normal model. Then, the two components of the model were fitted consecutively (Ovaskainen & Abrego, 2020). The analysis was restricted to the genera that were present in at least four samples within each treatment and host species, which resulted in 82 genera for AS NC models, 64 genera for AS LC models, 96 genera for CR NC models, and 63 genera for CR LC models. This stringent criterion was used as rare species lack adequate information for taxon‐specific modeling. As fixed explanatory variables in matrix X of HMSC, we included the categorical factor time, as well as the log‐transformed continuous variable of sequencing depth, which controlled for the variation in sequencing effort among samples. To account for the hierarchical study design, we included cage and individual ID random effects in the models. To examine whether the responses of the genera to time showed a phylogenetic signal, we included in the analysis a phylogenetic correlation matrix C among the genera, obtained as explained in the previous section. The phylogenetic signal is measured using the parameter *ρ*, which takes values from 0 to 1, a value of 0 meaning no phylogenetic signal in the response to time, and a value of 1 meaning a completely phylogenetically structured response to time. A significant positive (negative) association with T1 in the binomial model means that the genus has a higher (lower) probability of occurrence in T1. A significant association in the log‐normal model means that, when present, the genus is more (less) abundant in T1. The genera with a positive response to time in captivity with posterior probability of >0.9 were considered as significantly enriched in captivity. The genera with a negative response to time in captivity, with posterior probability <0.1 were considered as significantly enriched in nature. The posterior probability of >0.9 indicates that >90% of the parameter estimates of the posterior distribution are positive. The posterior probability of <0.1 indicates that <10% of the parameter estimates of the posterior distribution are positive (hence, >90% are negative). We fitted the models assuming the default priors and sampled the posterior distribution running four Markov Chain Monte Carlo (MCMC) chains, each of which was run for 37,500 iterations, of which 12,500 were discarded as burn‐in. We thinned by 100 to obtain a total of 250 posterior samples per chain and 1000 posterior samples in total. We ensured MCMC convergence by measuring the potential scale reduction factor (Tikhonov et al., 2020) for the beta parameters (measuring the response to time in captivity) and the *ρ* parameters (measuring phylogenetic signal in beta parameters).

## 3 RESULTS

**Figure 1:**
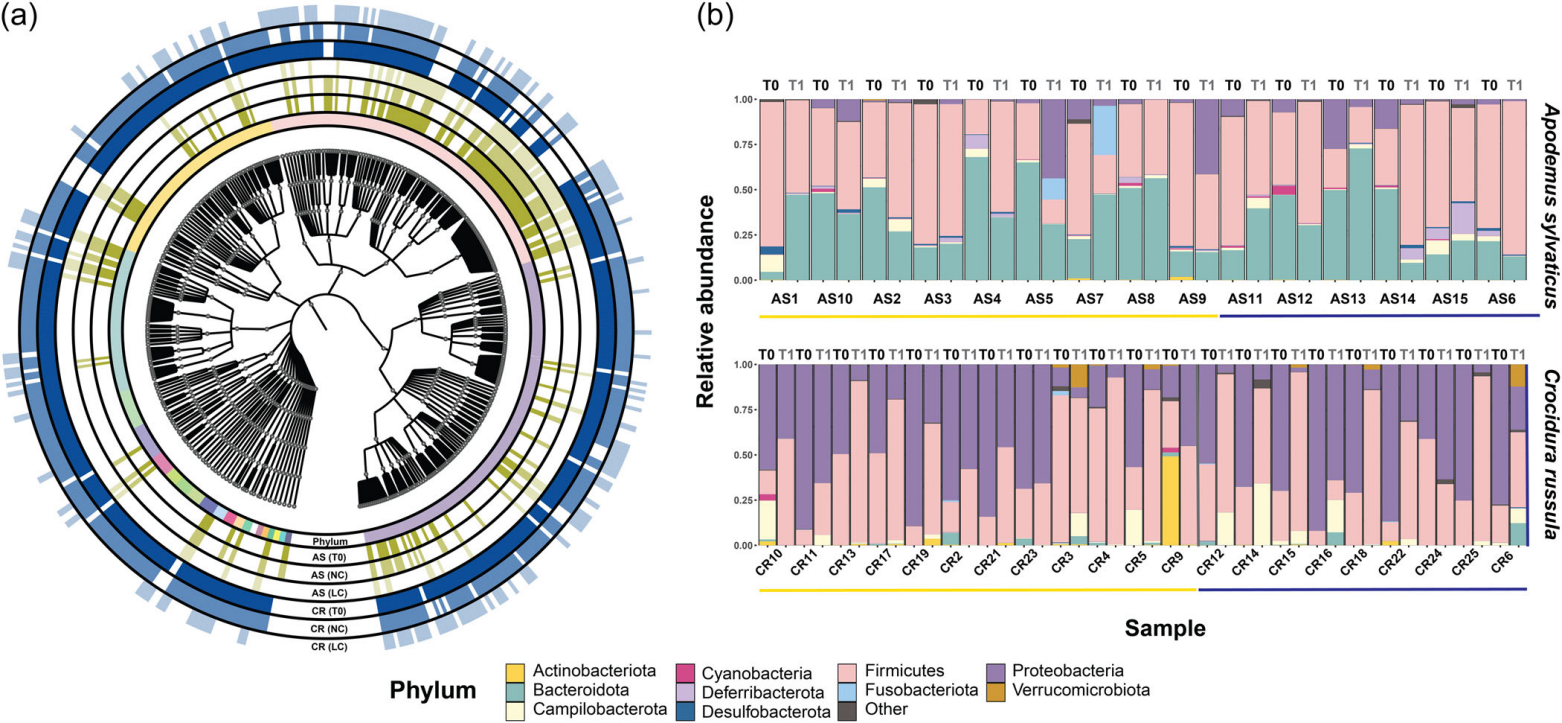
(a) Radial tree of life of presence/absence data at the genus level across all treatments indicating community level differences between treatments for both *Apodemus sylvaticus* (AS) and *Crocidura russula* (CR). Circular rings disseminate between Phylum, T0, natural conditions, and laboratory conditions for both species. (b) Stacked bar plots of sample pairs (T0 & T1) representing relative abundance at the community composition at the phylum level for natural conditions (yellow bar) and laboratory conditions (blue bar).

**Figure A2:**
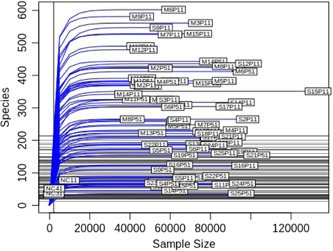
Species accumulation curves across all samples. All samples reached species asymptote.

We analyzed 72 fecal samples from 36 animals and four negative extraction controls to account for contamination. We generated 11,425,282 sequences (114,252 ± 42,356 per sample; mean and standard deviation, respectively) with a total of 6,570,726 (65,707 ± 21,936) sequences after quality filtering (for a full breakdown see Appendix A: Table A1). From these, 8176 unique amplicon sequence variants were generated (herein ASVs), which were assigned to 31 phyla, 68 classes, 142 orders, 226 families, and 427 genera (Figure 1). The 28 ASVs that were not assigned at least a bacterial Phylum annotation were removed from downstream analyses. Decontam detected 39 ASVs as contaminants, which were also removed from all parts of the analysis (Appendix A: Table A2). The ASV accumulation curves of all samples reached the asymptote, which confirmed sufficient sequencing depth to recover the complete microbial diversity (see Appendix A: Figure A2).

### 3.1 Characterization of wild microbiomes across time

**Figure 2:**
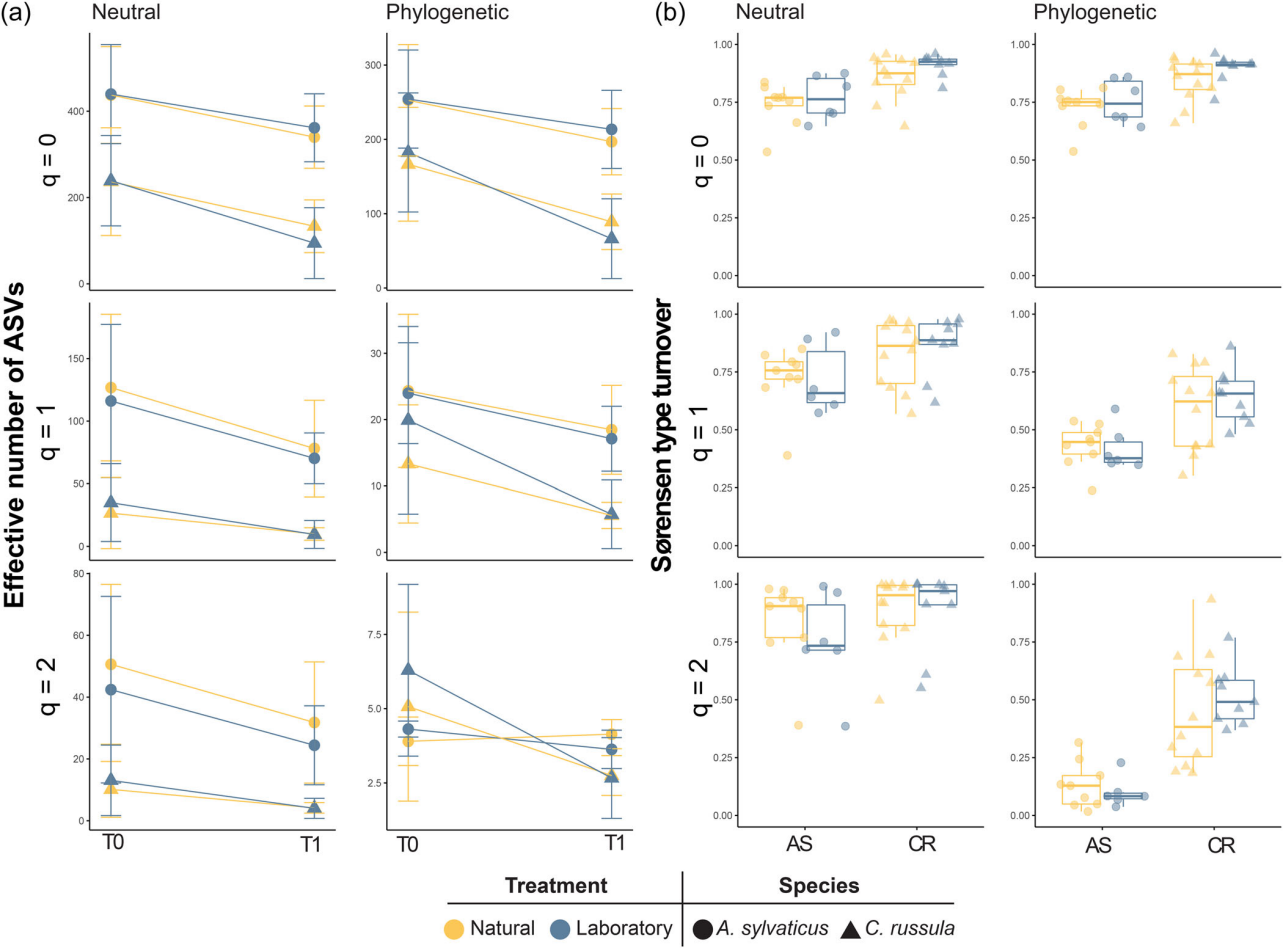
Treatment effects on neutral and phylogenetic Hill numbers calculated for alpha (A) and beta (B) diversity between treatments and time‐points. (a) Alpha diversity represented by the average difference from T0 to T1 for both *Apodemus sylvaticus* and *Crocidura russula* for each order of diversity and diversity dimension. (b) Beta diversity of data paired per individual animal for both *A. sylvaticus* (AS) and *C. russula* (CR) across the three orders of diversity and the two diversity dimensions.

Wild‐caught AS harbored a microbial community consisting of 12 phyla spanning 3299 ASVs (Figure 1). The microbiota was principally dominated by Firmicutes (51.6 ± 21.5%) and Bacteroidota (36 ± 20.7%), followed by Proteobacteria (5.7 ± 7.6%). In contrast, CR harbored a gut microbiota that consisted of 30 phyla spanning 2328 ASVs. The microbial communities were dominated by Proteobacteria (62.3 ± 23.8%) and Firmicutes (30.8 ± 21%), followed by Actinobacteria (2.6 ± 10.6%). As shown by the large standard deviations around means, the initial microbiome composition was highly variable across individuals captured in the wild (Figure 2a).

### 3.2 Effects of captivity and housing treatments

Hill numbers were calculated for three orders of diversity (*q* values) and across both neutral and phylogenetic measures yielding varying numbers of effective ASVs for AS and CR (Figure 2a). We observed a reduction in total detected ASVs for AS (*n* = 2302, −30.2%) and CR (*n* = 1126, −51.6%) across time. Further, we did not detect a significant interaction between time and treatment indicating that diversity loss after time in captivity occurred similarly in individuals from either treatment (CR_neutral_: *t*_*19*_ = −1.43, *p* = 0.16, CR_phylogenetic_: *t*_*19*_ = −1.71, *p* = 0.10, AS_neutral_: *t*_*13*_ = 0.11, *p* = 0.91, AS_phylogenetic_: *t*_*13*_ = −0.56, *p* = 0.58). Similarly, treatment had no significant effect on alpha diversity measured with either neutral or phylogenetic diversity metrics (CR_neutral_: *t*_*3*_ = −0.081, *p* = 0.941, CR_phylogenetic_: *t*_*3*_ = 0.207, *p* = 0.847, AS_neutral_: *t*_*2*_ = −0.274, *p* = 0.810, AS_phylogenetic_: *t*_*2*_ = 0.165, *p* = 0.884). However, we did detect a significant reduction in the alpha diversity between both time‐points using neutral diversity metrics for AS (*t*_*14*_ = −2.234, *p* = 0.042) and for CR (*t*_*20*_ = −4.138, *p* < 0.001); whereas when using phylogenetic diversity metrics, the difference between both time‐points was significant for CR (*t*_*20*_ = −4.861, *p* < 0.001), but not for AS (*t*_*14*_ = −1.862, *p* = 0.084).

We then calculated beta diversity between time points (Figure 2b), and observed that neither treatment (AS_neutral_: *t*_*2*_ = −0.284, *p* = 0.803, CR_neutral_: *t*_*3*_ = 0.416, *p* = 0.706) nor *q* value (AS_neutral_^q1^: *t*_28_ = −0.844, *p* = 0.406, AS_neutral_^q2^: *t*_28_ = 1.557, *p* = 0.131, CR_neutral_^q1^: *t*_40_ = −1.455, *p* = 0.154, CR_neutral_^q2^: *t*_40_ = 0.289, *p* = 0.774) had a significant effect on the dissimilarity between time points for both AS and CR using neutral diversity indices. Likewise, when using phylogenetic diversity measures, we did not detect a significant effect of treatment on dissimilarity between both time points (AS_phylogenetic_: *t*_*2*_ = −0.142, *p* = 0.900, CR_phylogenetic_: *t*_*3*_ = 1.151, *p* = 0.333). Interestingly, however, we detected that *q* value had a significant effect on reducing dissimilarity between time points when using the phylogenetic diversities (Figure 2b) for both AS (AS_phylogenetic_^q1^: *t*_28_ = −13.473, *p* > 0.001, AS_phylogenetic_^q2^: *t*_28_ = −26.784, *p* > 0.001) and CR (CR_phylogenetic_^q1^: *t*_40_ = −6.794, *p* > 0.001, CR_phylogenetic_^q2^: *t*_40_ = −10.231, *p* > 0.001) with higher *q* values resulting in lower dissimilarity between time‐points (all linear model results can be found in Appendix A: Table A3 a‐h).

**Figure 3:**
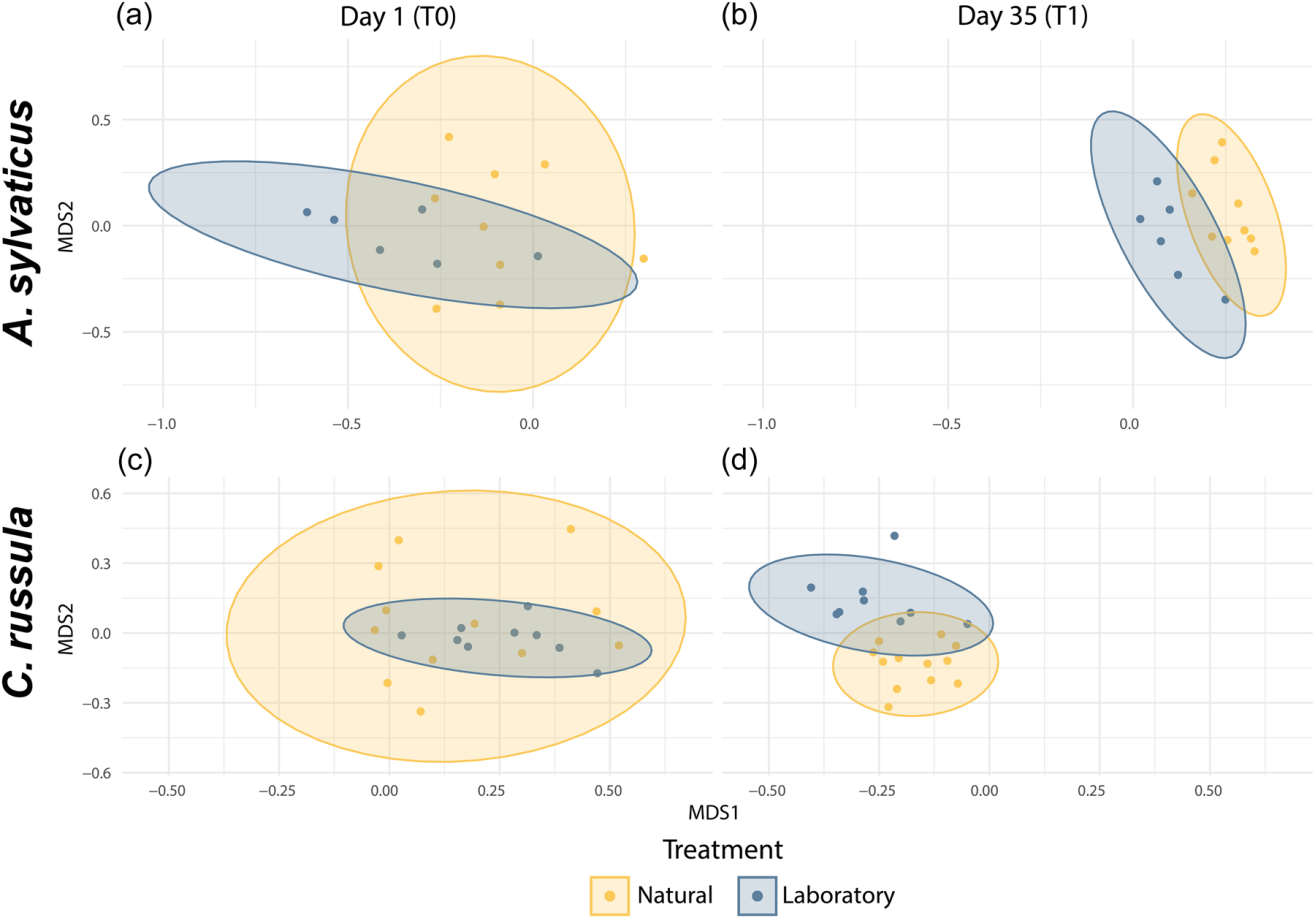
NMDS of community composition with 95% confidence intervals shaded in ellipses at Time point 1 (a, c): for *A. sylvaticus* and *C. russula* respectively representing the community composition of each individual immediately after being captured in the field (T0, day 1) and (b, d)**:** 35 days later (T1) representing the community composition diverge between treatments of Natural (yellow) and Laboratory (blue).

PERMANOVA analyses showed that while beta‐diversities were changing at similar rates and alpha diversities were showing similar decays, the composition between each treatment diverged after the captivity period (Figure 3). This separation was significant in CR as indicated by a significant interaction between treatment and time (*Pseudo‐F*_*1*_ = 5.171, *p* = 0.012, *R* ^2^ = 0.07), however, the same interaction was not significant in AS (*Pseudo‐F*_*1*_ = 1.297, *p* = 0.162, *R* ^2^ = 0.039), although some separation between treatments was visible after day 35 (Figure 3, Appendix A: Table A4).

**Figure 4:**
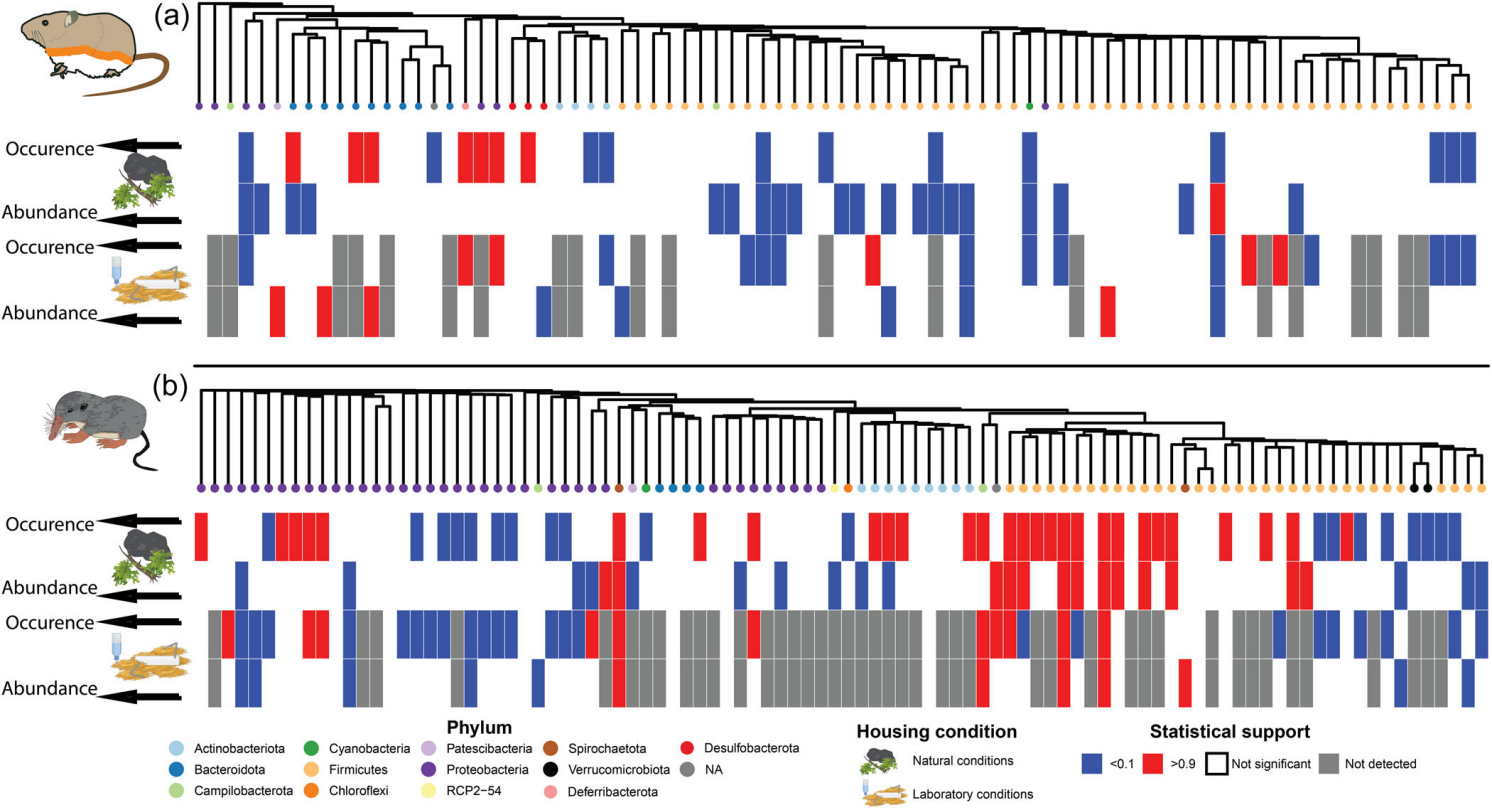
HMSC analysis showing the response of the most common genera to time in captivity, for (a) *Apodemus sylvaticus* and (b) *Crocidura russula*. Statistical support is provided in columns for both the presence‐absence/occurrence (binomial model) and abundance conditional on presence (lognormal model) models for natural conditions and laboratory conditions. Statistical support of >0.9 (red bars) was considered a significant increase of an ASV after 35 days; statistical support of <0.1 (blue bars) represents a significant decrease of an ASV; statistical support between 0.1 and 0.9 (white bars) was considered as not significantly affected by time in captivity (grey bars) represent depleted ASVs which were lost after 35 days. See the methods section for more details on how statistical support is interpreted in the Bayesian context.

We assessed the differential response of the most common genera to captivity in terms of probability of presence (binomial submodel of the Hurdle model) and log‐abundance conditional on the presence (lognormal submodel of the Hurdle model) (Table 2). We observed that 23% and 15% of the genera detected in AS showed a negative association with time in the binomial models in laboratory and natural conditions, respectively, whereas 8% and 9% of the genera showed positive associations (Figure 4). In the case of CR, 46% and 20% of common genera showed negative associations with time in the laboratory and natural conditions, respectively, while 25% and 30% showed positive associations. In AS abundance models, 8% and 24% of genera decreased and 6% and 1% increased in time, respectively under laboratory and natural conditions. In contrast, a predominance of negative associations was not as clear in CR abundance models: 11% and 14% of the genera showed a negative association with time whereas 8% and 14% showed positive associations, under laboratory and natural conditions. Of the few positive associations detected in the AS abundance models, we detected the proliferation of *Rikenella* (Phylum: Bacteriodota), *Odoribacter* (Phylum: Bacteriodota), Lachnoclostridium (Phylum: Firmicutes), and *Candidatus_Saccharimonas* ASVs which were solely detected in the LC treatment. In contrast, most positive associations in CR were with respect to genera belonging to Firmicutes and most negative associations with Bacteroidetes across both treatments. Additionally, we found some evidence that the NC housing conditions mitigated the loss of genus level diversity found at T0 which was not found in the T1 sample from the LC treatment (Figures 1a and 4), especially with CR. Loss of genera was not observed in the NC treatment which maintained all the detected genera from their initial day 1 sample (Figure 4), albeit with reduced alpha diversities and loss of ASVs. Moreover, these associations showed phylogenetic structure in both species as determined by the *ρ* values calculated by the HMSC model (see Table 2).

**Table 2:** HMSC results indicating the specific model used as well as the phylogenetic signal (*ρ* value) in species responses to time in captivity; higher *ρ* values indicate a higher phylogenetic signal and the 90% credible intervals not overlapping zero indicate strong evidence for its significance

Species	Treatment	Model	*ρ* Value	90% credible interval
*Apodemus sylvaticus*	NC	Binomial	0.68	[0, 0.98]
*Apodemus sylvaticus*	LC	Binomial	0.22	[0, 0.89]
*Apodemus sylvaticus*	NC	Lognormal	0.79	[0.08, 0.99]
*Apodemus sylvaticus*	LC	Lognormal	0.81	[0, 0.99]
*Crocidura russula*	NC	Binomial	0.9	[0.64, 1]
*Crocidura russula*	LC	Binomial	0.66	[0, 0.96]
*Crocidura russula*	NC	Lognormal	0.76	[0.08, 0.98]
*Crocidura russula*	LC	Lognormal	0.59	[0, 0.97]

## 4 DISCUSSION

Continued work in assessing the role of the gut microbiota on host fitness has demonstrated that the maintenance of a biologically optimal microbiota offers many benefits, not only to the host but also to the representativeness and translatability of research (Hauffe & Barelli, 2019; Hird, 2017). In the search for useful management practices to retain wild‐like gut microbial communities in animals taken into captivity, we measured the impact of two housing treatments in two small mammals and quantified several features of their associated microbial communities. We observed that increased exposure to environmental microorganisms did not significantly prevent gut microbiota diversity loss when compared to conventional experimental housing setups. Instead, our results showed that both treatments resulted in a reduction and restructuring of the gut microbiota community. However, the microbial composition changed in different directions between treatments in CR, indicating that microbial trajectories may be influenced by environmental factors in a species‐specific manner.

The relevance of environmental microbes is an important facet to consider when measuring host fitness as microbiota dysbiosis has been associated with factors that might reduce research translatability, such as immune function (Fujimura et al., 2014; Schuijt et al., 2016; Zhang et al., 2021), metabolism (Fan & Pedersen, 2021; Raymann & Moran, 2018; Sommer et al., 2016) and behavior (Davidson et al., 2020; Raulo et al., 2021; Singh et al., 2019). However, in our experiment, housing animals in a (semi) sterile environment or an environment enriched with natural elements had a similar effect on microbial alpha diversity patterns. This observation is in contrast with previous studies, which demonstrated that higher diversity in the environment does yield higher complexity in the host subsystem (Sbihi et al., 2019; Zhao et al., 2020; Zhou et al., 2016). Contrasting our studies, the main difference is likely due to the developmental stage of the analyzed animals, and the associated maturity of their gut microbial communities (Beura et al., 2016; Liu et al., 2021; Sbihi et al., 2019; Zhou et al., 2016). Our study included adults, which were shown to host a diverse microbial community before being introduced into captivity. In doing so, the starting gut composition was significantly more diverse compared to early‐life conspecifics (Nemergut et al., 2013). As such, the ecological niches within the adult gut‐microbiomes were likely already occupied (Langille et al., 2014; Turnbaugh et al., 2009) leading to competitive exclusion from the existing bacterial community (Baumgartner et al., 2021; Zmora et al., 2018). Thus, the lability of juvenile gut microbiomes may further promote the uptake of passively acquired environmental bacteria (Liu et al., 2021), while such a mode of acquisition seems to be negligible in adulthood, as suggested by the overall diversity loss in our experiment.

Despite the limited effect of the tested treatments at mitigating diversity loss, we did observe significant gut microbiota variation between time points in both species. Regarding alpha diversity, the observed patterns were somehow alike in the two species, even though the microbial communities associated with wild animals were radically different between AS and CR. Previous studies have demonstrated that captivity itself can significantly alter the gut microbiome of many species and may be due to a myriad of reasons, including access to nutrients, and changes in ambient temperatures/humidity (Sepulveda & Moeller 2020; Nicholls et al., 2016; Rosenbaum et al., 2009), diet (Bibbò et al., 2016; Martínez‐Mota et al., 2020; Morrison et al., 2020), which are all readily manipulated when entering captivity conditions and may also be confounded by evolutionary histories as species' responses to captivity can be host‐specific (Alberdi et al., 2021; Weinstein et al., 2021). Moreover, environmental complexity found in wild environments is not only a constituent of changes to the soil and physical surroundings but additionally to the many available bacteria in changing diets and water sources that were not tested in this experiment (Nyholm et al., 2022). As such, changing these conditions can lead to a wide range of responses, with studies reporting both significant microbial diversity increases and decreases in host responses to captivity (see [Alberdi et al., 2021] for further discussion).

Unlike overall diversity, the compositional response of the gut microbiotas to captivity and experimental treatments differed between species, which suggests that environmental access to microbes may have some effect in influencing the trajectory of the gut microbiome across different species. On the one hand, regardless of the treatment, we observed a significantly higher microbiota turnover in CR than in AS. By layering the diversity metrics at different orders of diversity with the phylogenetic information of each ASV, we were able to detect that in AS the main phylogenetic groups remained stable (Figure 2b). This indicates that when only using neutral diversity metrics, studies may omit valuable information as to whether the community changes are biologically meaningful signals or not. On the other hand, the CR gut microbiome demonstrated a clear interaction between time and treatment and separation of microbial composition based on housing conditions, which was not detected in AS. The lack of information on the microbial communities present in the natural elements prevented us to ascertain whether the observed variation was directly produced by the acquisition of environmental bacteria. However, the differences observed between AS and CR indicate that the response to the environment is likely to be highly specific‐specific and that many host‐specific factors may impact microbial sensitivity to environmental changes. We explored each host's response to the environment using HMSC, finding significant changes in the gut microbiota of specific genera. In most cases, genus‐level associations with time were either neutral or negative suggesting that the genera which responded to time in captivity were more likely to respond negatively, that is, decreasing abundances. Despite this, we did observe that some taxa proliferated under captive conditions. In AS, while only speculative, the proliferation of *Odoribacter* (Hiippala et al., 2020) has been known to increase propionate production, while additionally *Rikinella* has been known to increase lipid metabolism and energy regulation (Gálvez‐Ontiveros et al., 2020), potentially leading to positive effects on the gut health of AS. The severe drop in compositional turnover when increasing the order of diversity in phylogenetic metrics indicated that the replacement of ASVs mostly stemmed from closely related ASVs, rather than distant taxonomic groups. In contrast, we detected a highly significant, strong correlation with phylogeny in the CR data suggesting phylogenetically related genera were responding in the same way. Principally, both the presence and abundance of Proteobacteria genera significantly reduced, while bacteria within Firmicutes significantly increased in relative abundance (Figure 4b). A similar trend was found in *Suncus murinus* (Family: Soricidae), where domesticated individuals showed a significant reduction in the prevalence of Proteobacteria with the replacement of Firmicutes (Shinohara et al., 2019). These changes were hypothesized to have an important role in lactic acid fermentation and digestion of novel food types (Shinohara et al., 2019).

## 5 CONCLUSIONS

Our study showed that introducing natural elements into captivity conditions did not mitigate diversity loss in either species. Hence, considering the additional logistical burden, for example, time/resources spent in collecting housing materials, cleaning, and movement of materials compared to conventional materials which can be readily acquired, our results do not support the use of microbially enriched environments to retain wild‐like microbiotas in captivity. However, we observed that the natural elements triggered different compositional changes in different host species. Thus, implementing appropriate experimental caution through the use of pilot studies may be important when determining the suitability of microbially enriched environments for different species (Teijlingen & Hundley, 2002). Ultimately, our study shows that enriching captivity housing conditions with natural elements can shape the trajectories of microbiota variation and that this can happen in a species‐specific manner.

## AUTHOR CONTRIBUTIONS

**Adam Koziol**: Conceptualization (lead); formal analysis (equal); investigation (lead); methodology (lead); visualization (equal); writing–original draft (lead); Writing–review & editing (lead). **Iñaki Odriozola**: Data curation (equal); formal analysis (equal); methodology (supporting); supervision (equal); validation (equal); visualization (equal); writing–original draft (supporting); writing–review & editing (supporting). **Lasse Nyholm**: Investigation (supporting); methodology (supporting); validation (supporting); writing–original draft (supporting); writing–review & editing (supporting). **Aoife Leonard**: Data curation (supporting); formal analysis (supporting); validation (supporting); visualization (supporting); writing–review & editing (supporting). **Carlos San José**: Conceptualization (supporting); investigation (supporting); methodology (supporting); project administration (supporting); supervision (supporting); writing–review & editing (supporting). **Joana Pauperio**: Conceptualization (supporting); investigation (supporting); methodology (supporting); writing–review & editing (supporting). **Clara Ferreira**: Conceptualization (supporting); data curation (supporting); investigation (supporting); methodology (supporting); resources (supporting); writing–review & editing (supporting). **Anders J. Hansen**: Conceptualization (supporting); funding acquisition (supporting); methodology (supporting); project administration (supporting); supervision (supporting). **Ostaizka Aizpurua**: Conceptualization (supporting); formal analysis (supporting); investigation (supporting); methodology (supporting); project administration (supporting); supervision (supporting); writing–review & editing (supporting). **M. Thomas P. Gilbert**: Conceptualization (equal); funding acquisition (supporting); project administration (supporting); supervision (supporting); writing–original draft (supporting); writing–review & editing (supporting). **Antton Alberdi**: Conceptualization (lead); formal analysis (equal); funding acquisition (lead); investigation (supporting); methodology (lead); resources (equal); visualization (supporting); writing–original draft (supporting); writing–review & editing (equal).

## CONFLICT OF INTEREST

None declared.

## ETHICS STATEMENT

All animal captures and captivity experiments were approved by the Regional Government of Gipuzkoa under license codes PRO‐AE‐SS‐206 and PRO‐AE‐SS‐168. All experiments were performed following the agreed‐upon guidelines and regulations.

## Data Availability

Sequence data are publicly available under the ENA project accession number PRJEB48838: https://www.ebi.ac.uk/ena/browser/view/PRJEB48838

## APPENDIX A

See Figures A1 and A2 and Tables A1, A2, A3, A4.

**Table A1:** Quality filtering of raw sequence reads for each sample from initial reads to final ASVs

Sample ID	Number of initial reads	Average forward read length	Average reverse read length	Primer‐trimmed reads	Filtered reads	Dereplicated reads	ASVs before chimera filtering	ASVs after chimera filtering	Reads represented by ASVs
M10P11	96,790	295	275	96,760	89,468	37,739	5751	716	45,162
M10P51	91,760	296	277	91,728	85,274	32,003	3833	437	48,835
M11P11	106,689	295	270	106,655	99,491	36,867	3750	493	62,167
M11P51	88,216	293	271	88,186	78,282	31,903	3964	380	42,999
M12P11	96,046	297	274	96,030	91,282	37,884	5063	649	46,713
M12P51	87,990	297	276	87,967	83,048	29,080	3819	441	45,975
M13P11	117,929	298	283	117,888	112,196	30,464	6521	509	63,966
M13P51	118,706	291	285	118,636	100,215	26,278	6288	291	50,967
M14P11	80,167	292	284	80,153	72,480	23,798	4192	459	39,174
M14P51	138,320	294	285	138,252	126,374	39,586	3690	519	81,349
M15P11	170,094	292	269	169,999	145,340	67,001	5637	633	73,759
M15P51	144,609	299	270	144,572	137,022	54,434	4957	479	78,276
M1P11	99,687	295	258	99,650	92,718	42,457	3144	414	45,971
M1P51	95,687	293	260	95,655	86,242	34,400	4601	434	46,630
M2P11	98,285	298	281	98,257	91,386	31,780	5562	512	48,261
M2P51	126,529	292	270	126,432	106,270	38,399	5108	527	55,070
M3P11	155,555	292	259	155,460	134,782	58,773	4303	654	75,001
M3P51	195,103	299	258	195,059	185,803	73,456	7481	540	96,636
M4P11	171,195	292	268	171,140	148,959	30,904	5403	361	92,218
M4P51	126,073	295	268	126,031	116,108	45,108	5863	486	58,233
M5P11	184,764	298	269	184,716	175,335	68,721	12584	678	86,756
M5P51	99,766	298	271	99,732	94,799	28,252	3766	325	63,784
M6P11	125,702	296	269	125,655	117,567	53,559	4439	752	61,612
M6P51	162,691	297	271	162,632	154,614	35,780	4770	492	97,196
M7P11	105,769	298	271	105,725	100,303	39,491	3219	623	59,877
M7P51	136,513	299	273	136,456	130,339	32,911	7216	405	78,354
M8P11	173,262	297	270	173,210	164,496	65,348	9259	678	86,913
M8P51	88,098	295	270	88,062	82,147	32,280	4994	354	41,132
M9P11	95,276	292	277	95,233	80,071	36,244	2136	627	45,794
M9P51	104,545	293	277	104,519	91,983	29,223	3406	421	55,966
NC11	10,623	295	287	10,616	9933	1344	79	77	9722
NC21	2261	296	286	2260	2141	331	35	32	2057
NC31	2458	297	287	2456	2281	405	38	36	2182
NC41	2636	298	286	2636	2482	429	52	52	2363
S10P11	98,029	299	273	98,011	93,532	16,021	770	353	81,163
S10P51	99,296	298	269	99,279	94,523	15,421	2096	117	71,240
S11P11	109,643	296	284	109,625	104,145	14,293	1100	123	87,173
S11P51	88,572	297	284	88,563	83,145	15,096	1814	138	64,941
S12P11	200,073	297	272	200,029	189,851	61,947	13,792	711	98,834
S12P51	75,606	298	288	75,582	71,158	11,862	982	81	56,764
S13P11	123,606	295	271	123,562	115,738	24,895	4895	296	77,626
S13P51	132,630	298	287	132,600	124,585	30,115	5390	291	74,186
S14P11	206,199	291	284	206,100	174,331	45,232	10,733	482	95,321
S14P51	79,919	292	288	79,905	70,793	9971	573	57	62,209
S15P11	251,040	295	271	250,978	235,252	57,311	14,865	545	133,440
S15P51	81,859	294	287	81,837	75,538	9764	594	79	67,201
S16P11	132,078	293	272	132,056	122,795	19,900	2258	208	97,032
S16P51	93,866	295	285	93,837	87,783	19,051	2207	149	64,667
S17P11	158,046	296	285	158,007	148,823	39,037	7275	448	89,183
S17P51	147,398	297	287	147,359	139,227	35,712	6737	333	81,508
S18P11	142,135	292	272	142,072	123,741	25,602	5623	296	78,791
S18P51	66,957	299	266	66,942	63,922	8673	779	90	55,636
S19P11	125,308	299	283	125,279	120,485	20,238	2821	256	96,684
S19P51	97,326	298	284	97,280	90,831	15,987	2763	189	67,037
S21P11	155,087	299	258	155,047	148,938	34,548	7973	317	90,833
S21P51	181,482	298	259	181,432	173,139	40,657	7781	260	102,983
S22P11	99,774	291	282	99,683	80,449	13,890	2940	250	52,211
S22P51	134,685	292	284	134,630	117103	14474	3279	138	82610
S23P11	158,933	297	259	158,888	151,108	35,063	7768	276	89,170
S23P51	75,563	296	262	75,527	71,096	13,675	1652	84	53,711
S24P11	127,873	294	282	127,819	117,532	16,927	4691	277	81,625
S24P51	115,895	295	283	115,832	105,360	8103	718	86	95,863
S25P11	129,221	296	282	129,171	120,091	15,379	3513	230	86,775
S25P51	112,446	297	284	112,399	104,109	6841	451	57	95,201
S2P11	191,410	298	289	191,359	181,346	50,008	10,071	550	98,420
S2P51	101,890	291	288	101,842	85,542	14,052	1706	101	66,295
S3P11	76,301	293	290	76,274	69,598	15,010	851	454	58,783
S3P51	92,025	294	289	92,005	83,943	19,487	1835	344	63,776
S4P11	89,161	296	288	89,149	83,445	19,491	1517	432	64,439
S4P51	84,771	297	290	84,750	79,243	13,958	1863	82	58,796
S5P11	81,843	298	289	81,829	76,822	11,537	810	103	66,896
S5P51	85,355	298	286	85,342	79,864	19,654	2223	223	55,377
S6P11	122,178	291	283	122,116	100,890	20,432	2838	240	73,663
S6P51	98,257	292	286	98,241	86,641	24,916	2417	366	61,319
S9P11	132,306	294	283	132,271	119,276	40,263	4381	700	55,538
S9P51	80,321	295	285	80,304	73,721	14,730	1684	125	56,233

**Table A2:** ASVs detected by decontam—all ASVs were removed

ASVs	Kingdom	Phylum	Class	Order	Family	Genus
ASV_38	Bacteria	Proteobacteria	Gammaproteobacteria	Vibrionales	Vibrionaceae	*Vibrio*
ASV_126	Bacteria	Proteobacteria	Gammaproteobacteria	Burkholderiales	Comamonadaceae	*Delftia*
ASV_486	Bacteria	Firmicutes	Clostridia	Lachnospirales	Lachnospiraceae	*Cellulosilyticum*
ASV_803	Bacteria	Proteobacteria	Gammaproteobacteria	Enterobacterales	Morganellaceae	*Morganella*
ASV_829	Bacteria	Firmicutes	Clostridia	Clostridiales	Clostridiaceae	*Clostridium_sensu_stricto_13*
ASV_875	Bacteria	Firmicutes	Clostridia	Lachnospirales	Lachnospiraceae	*Herbinix*
ASV_1023	Bacteria	Actinobacteriota	Actinobacteria	Corynebacteriales	Dietziaceae	*Dietzia*
ASV_1207	Bacteria	Firmicutes	Clostridia	Clostridiales	Clostridiaceae	*Clostridium_sensu_stricto_13*
ASV_1318	Bacteria	Proteobacteria	Gammaproteobacteria	Pasteurellales	Pasteurellaceae	*NA*
ASV_1370	Bacteria	Firmicutes	Clostridia	Lachnospirales	Lachnospiraceae	*Epulopiscium*
ASV_2014	Bacteria	Firmicutes	Clostridia	Lachnospirales	Lachnospiraceae	*Anaerosporobacter*
ASV_2087	Bacteria	Firmicutes	Clostridia	Lachnospirales	Lachnospiraceae	*Lachnospiraceae_NK4A136_group*
ASV_2470	Bacteria	Firmicutes	Clostridia	Clostridiales	Clostridiaceae	*Clostridium_sensu_stricto_1*
ASV_2502	Bacteria	Proteobacteria	Gammaproteobacteria	Burkholderiales	Comamonadaceae	*Aquabacterium*
ASV_2556	Bacteria	Firmicutes	Clostridia	Lachnospirales	Lachnospiraceae	*Lachnospiraceae_NK4A136_group*
ASV_2913	Bacteria	Actinobacteriota	Actinobacteria	Corynebacteriales	Corynebacteriaceae	*Corynebacterium*
ASV_2959	Bacteria	Firmicutes	Bacilli	Staphylococcales	Staphylococcaceae	*Jeotgalicoccus*
ASV_3004	Bacteria	Firmicutes	Clostridia	Lachnospirales	Lachnospiraceae	*Lachnospiraceae_NK4A136_group*
ASV_3034	Bacteria	Proteobacteria	Gammaproteobacteria	Pseudomonadales	Moraxellaceae	*Acinetobacter*
ASV_3077	Bacteria	Firmicutes	Bacilli	Erysipelotrichales	Erysipelotrichaceae	*Turicibacter*
ASV_3220	Bacteria	Firmicutes	Bacilli	Staphylococcales	Gemellaceae	*Gemella*
ASV_3388	Bacteria	Proteobacteria	Gammaproteobacteria	Burkholderiales	Oxalobacteraceae	*Noviherbaspirillum*
ASV_3691	Bacteria	Proteobacteria	Alphaproteobacteria	Rhizobiales	Rhizobiaceae	*Allorhizobium‐Neorhizobium‐Pararhizobium‐Rhizobium*
ASV_3732	Bacteria	Firmicutes	Bacilli	Lactobacillales	Aerococcaceae	*Aerococcus*
ASV_4125	Bacteria	Proteobacteria	Gammaproteobacteria	Pseudomonadales	Pseudomonadaceae	*Pseudomonas*
ASV_4126	Bacteria	Proteobacteria	Alphaproteobacteria	Sphingomonadales	Sphingomonadaceae	*Sphingomonas*
ASV_4238	Bacteria	Proteobacteria	Gammaproteobacteria	Burkholderiales	Oxalobacteraceae	*Noviherbaspirillum*
ASV_4771	Bacteria	Actinobacteriota	Actinobacteria	Micrococcales	Micrococcaceae	*Renibacterium*
ASV_4846	Bacteria	Proteobacteria	Gammaproteobacteria	Burkholderiales	Oxalobacteraceae	*Massilia*
ASV_4930	Bacteria	Proteobacteria	Gammaproteobacteria	Burkholderiales	Comamonadaceae	*Acidovorax*
ASV_5194	Bacteria	Proteobacteria	Alphaproteobacteria	Azospirillales	Azospirillaceae	*Azospirillum*
ASV_5676	Bacteria	Proteobacteria	Gammaproteobacteria	Pseudomonadales	Moraxellaceae	*Enhydrobacter*
ASV_5975	Bacteria	Firmicutes	Bacilli	Staphylococcales	Staphylococcaceae	*Staphylococcus*
ASV_6063	Bacteria	Firmicutes	Clostridia	Clostridia_vadinBB60_group	NA	NA
ASV_6312	Bacteria	Proteobacteria	Alphaproteobacteria	Azospirillales	Azospirillaceae	NA
ASV_6315	Bacteria	Proteobacteria	Alphaproteobacteria	Caulobacterales	Caulobacteraceae	*Brevundimonas*
ASV_6507	Bacteria	Bacteroidota	Bacteroidia	Flavobacteriales	Weeksellaceae	*Chryseobacterium*
ASV_7211	Bacteria	Proteobacteria	Gammaproteobacteria	Enterobacterales	Enterobacteriaceae	NA
ASV_7452	Bacteria	Firmicutes	Clostridia	Lachnospirales	Lachnospiraceae	NA

**Table A3:** a–h: Linear mixed model results for each model

	Value	Std. error	*df*	*t* Value	*p* Value
*a. Crocidura neutral—beta diversity*					
(Intercept)	0.8689446	0.04057842	40	21.390725	0.000
TreatmentLC	0.0242597	0.05860281	3	0.415690	0.7056
qvalueq. 1	−0.037708	0.02566214	40	−1.454951	0.1535
qvalueq. 2	0.0070877	0.02566214	40	0.289183	0.7739
*b. Crocidura phylogenetic—beta diversity*					
(Intercept)	0.8420088	0.03970625	40	21.205950	0
TreatmentLC	0.0582521	0.05058904	3	1.151476	0.333
qvalueq. 1	−0.2577344	0.03793803	40	−6.793564	0
qvalueq. 2	−0.3881623	0.03793803	40	−10.231485	0
*c. Apodemus neutral—beta diversity*					
(Intercept)	0.7585985	0.04716536	28	16.083807	0
TreatmentLC	−0.0192982	0.06797048	2	−0.283920	0.8032
qvalueq. 1	−0.0283791	0.03361145	28	−0.844327	0.4056
qvalueq. 2	0.0523375	0.03361145	28	1.557134	0.1307
*d. Apodemus phylogenetic—beta diversity*					
(Intercept)	0.7407412	0.02745100	28	26.984119	0
TreatmentLC	−0.0053725	0.03792140	2	−0.141674	0.9003
qvalueq. 1	−0.3116469	0.02313081	28	−13.473241	0
qvalueq. 2	−0.6195252	0.02313081	28	−26.783553	0
*e. Crocidura neutral—alpha diversity*					
(Intercept)	5.394585	0.19493481	82	27.67379	0
FirstorSecondT2	−0.822103	0.19865859	20	−4.13827	0.0005
TreatmentLC	−0.020447	0.25226285	3	−0.08105	0.9405
qvalueq. 1	−2.451099	0.06864411	82	−35.70734	0
qvalueq. 2	−3.293441	0.06864411	82	−47.97850	0
*f. Crocidura phylogenetic—alpha diversity*					
(Intercept)	5.032623	0.16111364	80	31.23648	0
FirstorSecondT2	−0.826753	0.17007061	20	−4.86123	0.0001
TreatmentLC	0.042234	0.20433123	3	0.20669	0.8471
qvalueq. 1	−2.534690	0.09074075	80	−27.93331	0
qvalueq. 2	−3.475121	0.09074075	80	−38.29725	0
FirstorSecondT2:qvalueq. 1phy	−0.092760	0.12832680	80	−0.72284	0.4719
FirstorSecondT2:qvalueq. 2phy	0.194257	0.1279426	80	1.4698	0.1455
*g. Apodemus neutral—alpha diversity*					
(Intercept)	6.160106	0.15415349	58	39.96086	0
FirstorSecondT2	−0.399795	0.17894850	14	−2.23413	0.0423
TreatmentLC	−0.050041	0.18263856	2	−0.27399	0.8098
qvalueq. 1	−1.501942	0.08511615	58	−17.64579	0
qvalueq. 2	−2.534178	0.08511615	58	−29.77318	0
*h. Apodemus phylogenetic—alpha diversity*					
(Intercept)	5.487686	0.08767164	56	62.59363	0
FirstorSecondT2	−0.206698	0.11099538	14	−1.86222	0.0837
TreatmentLC	0.016139	0.09767066	2	0.16524	0.8839
qvalueq. 1	−2.397517	0.06886942	56	−34.81251	0
qvalueq. 2	−4.107089	0.06891415	56	−59.60574	0
FirstorSecondT2:qvalueq. 1phy	−0.056658	0.09739606	56	−0.59338	0.5553
FirstorSecondT2:qvalueq. 2phy	0.176851	0.09739606	56	1.81579	0.0748

**Table A4:** PERMANOVA results for *A. sylvaticus* and *C. russula*

Main_effects	*df*	SumOfSqs	*R* ^2^	*F*	Pr(>*F*)
*A. sylvaticus*					
Treatment	1	0.756	0.083	2.853	0
First or second	1	1.117	0.123	4.214	0
Treatment: first or second	1	0.344	0.038	1.297	0.162
Residual	26	6.891	0.757	NA	NA
Total	29	9.108	1	NA	NA
*C. russula*					
Treatment	1	1.035	0.083	5.235	0
First or second	1	2.918	0.234	14.76	0
Treatment: first or second	1	1.022	0.082	5.171	0.012
Residual	38	7.513	0.602	NA	NA
Total	41	12.488	1	NA	NA
